# Clinical Profiles of Hospitalized Middle Eastern Patients With Nonvalvular Atrial Fibrillation: Analysis From the JoFib Study

**DOI:** 10.1155/ijvm/6426995

**Published:** 2026-08-07

**Authors:** Abdullah Al-kasasbeh, Ahmad Abdalmajeed Alghzawi, Amr Alfakhouri, Nazih Kadri, Ammar Aloqaily, Mohammad Al-oakily, Baha′ Awad, Ayman J. Hammoudeh

**Affiliations:** ^1^ Cardiology Section, Department of Internal Medicine, Faculty of Medicine, Jordan University of Science and Technology, Irbid, Jordan, just.edu.jo; ^2^ Ministry of Health, Amman, Jordan, behdasht.gov.ir; ^3^ Department of Internal Medicine, Dr. Sulaiman Al Habib Hospital, Buraidah, Alqassim, Saudi Arabia; ^4^ Nazih Kadri, Electrophysiology and General Cardiology Sections, Cardiology Department, Abdali Hospital, Amman, Jordan; ^5^ Department of Internal Medicine, Faculty of Medicine, Jordan University of Science and Technology, Irbid, Jordan, just.edu.jo; ^6^ Department of Internal Medicine, Faculty of Medicine, Mutah University, Karak, Jordan, mutah.edu.jo; ^7^ Department of Public Health and Community Medicine, Faculty of Medicine, Jordan University of Science and Technology, Irbid, Jordan, just.edu.jo; ^8^ Cardiology Department, Istishari Hospital, Amman, Jordan, istisharihospital.com

**Keywords:** atrial fibrillation, hospitalized, middle eastern population, new onset

## Abstract

**Background:**

Atrial fibrillation (AF) is the most prevalent sustained arrhythmia and is associated with significant morbidity and mortality. Data on AF in hospitalized patients in the Middle East are limited.

**Methods:**

We analyzed 536 hospitalized patients with nonvalvular AF from the Jordan atrial fibrillation (JoFib) registry. A prospective multicenter study of 2020 patients enrolled between May 2019 and December 2020. Patients were stratified into new‐onset atrial fibrillation (NOAF) and previously diagnosed AF, and their clinical characteristics and in‐hospital outcomes were compared.

**Results:**

The cohort had a mean age of 70.2 ± 14.1 years, and 48.7% were male. The most common comorbidities were hypertension (77.6%), diabetes (52.1%), and heart failure (31.3%). NOAF patients were significantly younger and more likely to be smokers, whereas those with prior AF had more comorbidities and larger left atrial size. Overall, in‐hospital mortality was 6.7% but was significantly higher in NOAF patients.

**Conclusions:**

Hospitalized patients with NOAF are younger, more often smokers, and face higher in‐hospital mortality compared with chronic AF, underscoring the need for early recognition and risk factor modification to prevent hospitalization.

**Trial Registration:**

ClinicalTrials.gov Identifier: NCT03917992.

**Clinical Perspective**

What is known:

• Atrial fibrillation (AF) is a common arrhythmia associated with increased morbidity and mortality, especially among hospitalized patients.
• New‐onset atrial fibrillation (NOAF) during hospitalization is associated with acute illness and remains under‐studied in the Middle East.

What the study adds:

• This is the first study from Jordan to characterize clinical differences between patients with NOAF and chronic AF in a hospitalized cohort.
• Patients with NOAF were significantly younger and more likely to be smokers, yet they had a higher in‐hospital mortality rate compared to those with chronic AF.
• These findings highlight the need for early identification and tailored management strategies for NOAF.

## 1. Introduction

Although the global prevalence of atrial fibrillation (AF) continues to rise alongside an aging population, the burden of this arrhythmia is particularly acute in the inpatient setting [1]. Notably, hospitalized patients with cardiovascular disease exhibit heightened vulnerability to developing AF, either as a primary diagnosis or as a complication during their hospital stay [2].

The impact of AF extends beyond its occurrence, significantly impairing patients′ quality of life through symptoms such as palpitations, fatigue, and shortness of breath [3]. Furthermore, the fear of its complications is well founded, as AF is associated with an elevated risk of mortality, primarily driven by thromboembolic events like stroke and the development of heart failure, contributing to a substantial number of deaths annually [3]. Several underlying conditions, including coronary artery disease, heart failure, diabetes, obesity, lipid metabolism disorders, and hypertension, have been consistently linked to an increased risk of developing AF [4].

There is little data on hospitalized patients with AF in the middle east. Literature from the region did not address hospitalized patients specifically [5–7]. The Jordan atrial fibrillation (JoFib) study, a nationwide prospective registry enrolling 2020 participants between May 2019 and October 2020, with a 1‐year follow‐up, provided crucial insights into the regional prevalence and management of this common arrhythmia. Notably, AF occurring during hospitalization frequently coincides with significant comorbidities and advanced cardiovascular dysfunction, a combination associated with a high incidence of adverse outcomes. This underscores the critical need to identify and proactively manage these patients in the outpatient setting, potentially mitigating the necessity for future hospital admissions. Building upon this understanding, our study focuses on thoroughly examining patients with new‐onset atrial fibrillation (NOAF) who were either admitted due to AF or developed AF during hospitalization for another reason, pinpointing specific clinical profiles at their initial presentation that can help identify individuals at higher risk, allowing for more focused outpatient care aimed at preventing future hospitalizations.

## 2. Methods

The JoFib study methods were previously described [8, 9]. In brief, this is a prospective observational registry that enrolled adults diagnosed with AF in multiple centers across Jordan. Only hospitalized patients with nonvalvular AF were included in this final analysis, which totaled 536.

Patients were categorized into two groups: chronic AF (patients diagnosed with AF before enrollment in the study) and NOAF (patients diagnosed with AF at the time of enrollment). Patients who had a prosthetic heart valve or evidence of at least moderate rheumatic valve disease were classified as having valvular AF and were subsequently excluded from our current study. Upon enrollment, baseline data were collected, including clinical and demographic profiles, laboratory data, EKG, transthoracic echocardiographic features, and the use of OACs and other pharmacological medications. Baseline CHA_2_DS_2_‐VASc and HAS‐BLED scores were calculated for all patients based on standard definitions [10, 11]. In‐hospital mortality from any cause was recorded. In our study, follow‐up was limited to the duration of hospitalization. The JoFib study was registered at clinicaltrials.gov (unique identifier number NCT03917992).

The JoFib study was approved by the Institutional Review Board (IRB) of participating centers. The study was conducted in accordance with the Declaration of Helsinki, and written informed consent was obtained from all participants at the time of enrollment in the registry.

### 2.1. Statistical Analysis

Continuous data were reported as means accompanied by standard deviations (SDs), whereas categorical parameters were presented as frequencies and percentages. To evaluate differences between the two study groups, we used independent‐sample *t*‐tests for means and chi‐square tests for categorical data. A *p* value of < 0.05 was considered statistically significant.

## 3. Results

A total of 536 patients were included in the analysis; their baseline characteristics are presented in Table 1. The study population comprised 48.7% males, with a mean age of 70.24 ± 14.1 years and a mean body mass index (BMI) of 29.55 ± 6.26 kg/m^2^. Overall, 14.7% of the cohort were current smokers.

**Table 1:** Patient clinical characteristics.

	Newly diagnosed *N* = 187	Previously diagnosed *N* = 349	Total *N* = 536	*p*
Gender (male)	94 (50.8)	167 (47.6)	261 (48.7)	0.477
Age mean (SD)	66.62 (15.96)	72.18 (12.61)	70.24 (14.1)	< 0.0001
BMI	29.5 (6.14)	29.57 (6.35)	29.55 (6.26)	0.905
Current smoker	35 (18.9)	44 (12.5)	79 (14.7)	0.047
Comorbidities				
DM	96 (51.9)	183 (52.1)	279 (52.1)	0.957
HTN	131 (70.8)	285 (81.2)	416 (77.6)	0.006
Dyslipidemia	92 (49.7)	169 (48.1)	261 (48.7)	0.728
Heart failure	44 (23.8)	124 (35.3)	168 (31.3)	0.006
CAD	32 (17.3)	56 (16)	88 (16.4)	0.69
History of stroke	24 (13)	68 (19.4)	92 (17.2)	0.062
COPD	11 (5.9)	18 (5.1)	29 (5.4)	0.691
Thyroid disease	10 (5.4)	38 (10.8)	48 (9)	0.037
Chronic kidney disease	24 (13)	61 (17.4)	85 (15.9)	0.184
Sleep apnea	3 (1.6)	15 (4.3)	18 (3.4)	0.105
CHA₂DS₂‐VASc	3.65 (2.035)	4.31 (1.758)	4.08 (1.885)	< 0.0001
HAS‐BLED	1.75 (1.1)	2.19 (1.188)	2.03 (1.178)	< 0.0001
Echo findings				
Left atrial size	4.15 (0.640)	4.30 (0.601)	4.25 (0.617)	0.012
Ejection fraction	52.71 (13.135)	50.49 (12.637)	51.28 (12.823)	0.068
Left ventricular hypertrophy (477)	52 (32.3)	115 (36.4)	167 (35.0)	0.375
Pulmonary hypertension (532)	33 (18.1)	87 (24.9)	120 (22.6)	0.078
Mortality	18 (9.7)	18 (5.1)	36 (6.7)	0.043

Abbreviations: BMI, body mass index; CAD, coronary artery disease; COPD, chronic obstructive pulmonary disease; DM, diabetes mellitus; HTN, hypertension.

The most prevalent comorbidities were hypertension (77.6%), diabetes mellitus (52.1%), dyslipidemia (48.7%), and heart failure (31.3%). Other notable conditions included coronary artery disease (16.4%), prior stroke (17.2%), thyroid disorders (9.0%), chronic kidney disease (15.9%), chronic obstructive pulmonary disease (COPD) (5.4%), and obstructive sleep apnea (3.4%).

Several reasons led to patients being admitted, but mainly they were admitted because of noncardiovascular conditions (30.6%), AF (28.7%), heart failure (14.6%), and acute coronary syndrome (13.1%). Less frequent causes of admission included cerebrovascular accident (7.3%), bleeding events (3.4%), systemic embolization (excluding the brain) (1.3%), respiratory illnesses including COPD (0.9%), and syncope (0.2%).

The most reported symptoms of AF were palpitations (42.5%), dyspnea (29.1%), fatigue (17.2%), and dizziness (11.2%). Less frequent symptoms included syncope (1.5%) and chest pain (1.3%), whereas 37.3% of patients were asymptomatic.

Echocardiographic findings are detailed in Table 1. The mean left atrial diameter was 4.25 ± 0.62 cm, and the mean left ventricular ejection fraction was 51.28*%* ± 12.82*%*. Left ventricular hypertrophy was present in 35% of patients, and pulmonary hypertension in 22.6%.

In‐hospital mortality occurred in 6.7% of patients. Among those who died, the most common cause was sepsis (47.2%), followed by stroke (22.2%), acute myocardial infarction (8.3%), heart failure or cardiogenic shock (8.3%), and upper gastrointestinal bleeding (8.3%). Less frequent causes of death included respiratory failure (2.8%) and pulmonary embolism (2.8%).

The study population was stratified into two groups based on the timing of AF diagnosis: newly diagnosed and previously diagnosed cases. Baseline characteristics of the two groups are presented in Table 1. Although most variables did not differ significantly between the groups, some notable differences were observed. Patients with newly diagnosed AF were significantly younger on average but had a higher prevalence of smoking. In contrast, previously diagnosed patients had significantly higher rates of hypertension and heart failure. Both the HAS‐BLED and CHA₂DS₂‐VASc scores were significantly higher among patients with a prior diagnosis of AF, and they also had a larger left atrial diameter. Notably, in‐hospital mortality was unexpectedly higher among patients with newly diagnosed AF.

## 4. Discussion

Despite the growing prevalence of AF worldwide and its burden on healthcare systems, there remains a shortage of studies on the clinical profiles of hospitalized AF patients in the Middle East. This lack of regional data limits our ability to develop strategies tailored to this population [5–7, 12–16]. NOAF during hospitalization is frequently multifactorial, often occurring in the context of acute medical conditions such as sepsis or decompensated heart failure [2, 17]. It poses both diagnostic and therapeutic challenges due to the associated risks of thromboembolism and hemodynamic instability [2, 17]. Characterizing the clinical profiles of those who develop incidental in‐hospital AF enables early risk stratification, informs timely management, and supports targeted outpatient follow‐up to reduce readmissions and adverse events. In our cohort of 536 Jordanian inpatients, we delineated key features associated with NOAF.

Patients with NOAF were significantly younger than those with preexisting AF—a finding echoed by several studies comparing hospital‐onset versus chronic AF [18–21]. However, some evidence suggests the opposite trend [22], and others found no significant age‐related differences between the groups [23, 24]. We found no sex imbalance between groups, consistent with most reports [19–23], despite occasional reports of female predominance in chronic AF cohorts [18, 24]. These observations highlight possible population‐specific characteristics in NOAF presentation and risk factors.

In our cohort, smoking was significantly more prevalent among patients with newly diagnosed AF compared to those with preexisting AF, potentially reflecting nicotine′s proarrhythmic effects and its role in atrial remodeling. However, prior studies have reported no significant differences in smoking status between groups [19, 20, 22]. These divergent findings suggest that smoking may exert a population‐specific influence on the development of NOAF and merit further investigation.

Hypertension and heart failure occurred more frequently in the chronic AF group, mirroring reports of higher rates of HTN, HF, coronary artery disease, cerebrovascular disease, and prior stroke in preexisting AF [18, 19, 21, 22, 24]. Conversely, Massera et al. [18] noted greater myocardial infarction and malignancy prevalence in NOAF, and Zhang et al. [12] described increased HTN in ICU‐onset AF. Arrigo et al. [20] observed no HTN/HF difference but higher valve disease and thyroid disorders in chronic AF. Together, these data suggest baseline comorbidity underpins chronic AF, whereas acute illness severity drives NOAF.

We found that CHA₂DS₂‐VASc and HAS‐BLED risk scores were higher in chronic AF patients, indicating cumulative vascular risk—findings aligned with previous analyses [18, 21]. By contrast, severity indices such as APACHE III peaked in NOAF, underscoring the link between critical illness and incident AF [21, 23].

Using echocardiogram findings, we noticed that left atrial diameter was larger in chronic AF, consistent with long‐term remodeling, whereas left ventricular ejection fraction did not differ between groups. Similar atrial enlargement has been noted [19, 22], though some studies report reduced LVEF at NOAF onset, possibly reflecting acute hemodynamic compromise [24].

In our study, in‐hospital mortality was significantly higher among patients with newly diagnosed AF compared to those with a prior diagnosis. Several studies reported similar findings [12, 18, 20, 23–25], demonstrating that NOAF is associated with increased in‐hospital, ICU, or short‐term mortality. In contrast, other studies reported no significant difference in mortality between groups [19, 22]. Despite such variability, the overall trend across contemporary evidence supports our observation that NOAF is linked to a higher risk of in‐hospital death.

Regional data on AF in the Middle East region remain limited, particularly regarding in‐hospital NOAF. The Gulf SAFE registry, which included patients with AF from six Gulf countries, found that first‐time AF accounted for 37% of all AF presentations [26].More recently, the FLOW‐AF registry, a large, multicenter study of patients with newly diagnosed nonvalvular AF across the broader MENA region, reported a younger patient population with lower mean baseline CHA₂DS₂‐VASc and HAS‐BLED scores than those typically observed in Western cohort [6]. In comparison with the FLOW‐AF dataset, the present Jordanian cohort of hospitalized patients exhibited an older NOAF population and higher CHA₂DS₂‐VASc and HAS‐BLED scores, highlighting potential demographic, clinical, or environmental differences specific to Jordan.

### 4.1. Strengths and Limitations

To our best knowledge, our study is the first in Jordan and the Middle East to characterize specific clinical profiles of patients who develop AF during hospitalization with no prior documented history of AF, offering the first regional benchmark for hospital‐onset versus chronic AF. However, the study has limitations. Our analysis only covered the hospital stay, so long‐term outcomes of patients with NOAF were not studied. Furthermore, we did not collect detailed data on specific acute triggers for NOAF.

### 4.2. Clinical Implications

As the first study to characterize NOAF in Jordan, our work provides crucial, population‐specific clinical profiles. We found that patients with NOAF were younger and more often smokers yet faced significantly higher in‐hospital mortality. This challenges the reliance on traditional, Western‐derived risk profiles, which may underestimate the immediate risk in our demographic. These findings highlight a deceptively high‐risk phenotype, urging clinicians to adopt a heightened vigilance for younger, smoking patients during hospitalization for critical illnesses and to prioritize management of the acute precipitating illness. Future studies should validate findings across diverse Middle Eastern populations, investigate mechanisms behind unique traits, and evaluate tailored management strategies. Exploring genetic and environmental factors, alongside long‐term follow‐up, is critical to understanding NOAF progression and improving outcomes.

## 5. Conclusion

This study showed that hospitalized patients with NOAF are younger and more likely to be smokers than those with chronic AF. More importantly, they have a higher risk of in‐hospital mortality. Consequently, recognizing patients at risk of AF and initiating early intervention is of paramount importance.

**Nomenclature**
JoFib study: Jordan atrial fibrillation study
NOAF: new‐onset atrial fibrillation

## Funding

No funding was received for this manuscript.

## Disclosure

The authors have nothing to report.

## Ethics Statement

The study was conducted in accordance with the Declaration of Helsinki. Ethical approval was obtained from The Istishari Hospital IRB committee (No. IH.IRB2020/12AH).

## Conflicts of Interest

The authors declare no conflicts of interest.

## Data Availability

The data that support the findings of this study are available from the corresponding author upon reasonable request.
